# Efficacy of Brentuximab Vedotin and Nivolumab in Refractory or Relapsed Hodgkin Lymphoma: A Systematic Review

**DOI:** 10.7759/cureus.23452

**Published:** 2022-03-24

**Authors:** Sharina C Macapagal, Hayoung Lee, Javaria Abdul Jabbar, Anna Caroline Fjorden, Irene Tresa Joseph, Ramanpreet Kaur, Jihan A Mostafa

**Affiliations:** 1 Research, California Institute of Behavioral Neurosciences & Psychology, Fairfield, USA; 2 Psychiatry, California Institute of Behavioral Neurosciences & Psychology, Fairfield, USA

**Keywords:** immune-checkpoint inhibitors, antibody-drug conjugate, refractory and relapsed hodgkin lymphoma, nivolumab, brentuximab vedotin, immunotherapy

## Abstract

The central dilemma in treating patients with refractory or relapsed classical Hodgkin lymphoma (RRHL) is the developed resistance to chemotherapy. In recent years, significant advances have been made with the introduction of targeted immunotherapy such as brentuximab vedotin (BV) and nivolumab (NV). As monotherapy, BV and NV have demonstrated high response rates but with an opportunity for disease progression. In other studies, BV or NV is given in combination with chemotherapy as a bridge to hematopoietic stem cell transplantation for curative therapy. This review will investigate the effect of BV and NV as single agents, in combination with each other, or given concurrently with chemotherapy on the response and survival rate of patients with RRHL.

## Introduction and background

Hodgkin lymphoma (HL) is the proliferation of abnormal B lymphocytes which accounts for 15-20% of all lymphomas in some Western countries, with 90% constituting the classic subtype [1]. The standard treatment for HL is chemotherapy, which when combined with radiotherapy can cure up to 80% of patients [2]. However, about 10-30% of treated patients do not respond to the first-line therapy [3]. Refractory HL is defined as lymphomas that progress at any time during first-line chemoradiotherapy or occurrence of relapse three months after the completion of the treatment. Another important issue regarding treatment is the relapse of HL which is defined as the regrowth of more than 50% increase in the size or positivity of an initial negative positron emission tomography (PET) scan after a year of complete remission [4]. These patients are treated with salvage regimens such as high-dose chemotherapy (HDCT) and autologous stem cell transplantation (ASCT), which may cure approximately 50% of patients [2,3]. The long-term remission rate is 40-50% in relapsed patients and 25-30% in primary refractory disease [4]. Despite their effectiveness, these treatments have untoward long-term side effects such as cardiac and pulmonary toxicity, which are concerning for a few patients and therefore the providers as well. On the contrary, these post-treatment issues make these options unfavorable for already frail and elderly patients with multiple comorbidities.

Patients with refractory and relapsed Hodgkin lymphoma (RRHL) with failed multiple therapies, including multi-agent chemotherapy and radiotherapy, represent a therapeutic dilemma. The treatment goal for next-line treatment is long-term disease control with manageable adverse reactions. The antibody-drug conjugate brentuximab vedotin (BV) and immune checkpoint inhibitors (ICIs) such as nivolumab (NV) have been approved by the US Food and Drug Administration (FDA) as emerging treatments for RRHL [5]. BV has been approved for patients with RRHL who have failed ASCT as well as for those with consolidation after ASCT in high-risk patients. NV and pembrolizumab are approved for RRHL after ASCT and BV failure. The high effectiveness and low toxicity of immunotherapy with prolonged remission or disease stabilization make it a promising new treatment option for RRHL [5]. However, because it may be a comparatively newer treatment option, it is difficult to demonstrate its benefit as first-line monotherapy, especially because the response rates for standard therapies are more than 90% for most HLs [5,6].

According to Vassilakopoulos et al., BV is progressively being incorporated into the first-line treatment protocol of HL, while similar applications of NV and pembrolizumab appear promising [7]. In this review, the outcomes of immunotherapy treatment as a single agent or combined with chemotherapy or another targeted therapy agent will be reported. BV and NV are exclusively discussed because they are the foremost immunotherapy approved by the FDA utilized in RRHL.

## Review

Methodology

Protocol

We conducted a systematic review of 14 studies following the guidelines of the Preferred Reporting Items for Systematic Review and Meta-analysis (PRISMA) framework [8].

Eligibility Criteria

Two independent reviewers evaluated titles and abstracts of the articles included which were published in the last five years between 2016 and 2021. All clinical studies such as randomized controlled trials, case reports, and case series were included. The eligibility criteria included the following: (1) The study included patients diagnosed with RRHL after failing multiple lines of therapy. (2) The study intervention was BV or NV (both as a single agent or in combination with chemotherapy or another immunotherapy). (3) The study reported relevant endpoints (objective response rate, ORR; complete response, CR; progression-free survival, PFS; overall survival, OS).

Search Strategy

This integrative review searched for articles indexed within the PubMed database up to July 19, 2021, utilizing Medical Subject Headings (MeSH) terms and regular search keywords such as “Brentuximab vedotin,” “Nivolumab,” and “Refractory and Relapsed Hodgkin Lymphoma,” which were used both individually and in combination. Articles were then further screened to examine their relevance to the main goal of this study. The results of the search strategy using regular keywords are demonstrated in Table 1.

**Table 1:** Database search results with regular keywords.

Regular keywords	Total articles	Total articles after application of inclusion/exclusion criteria
Immunotherapy and refractory and relapsed Hodgkin Lymphoma	774	84
Brentuximab and refractory and relapsed Hodgkin Lymphoma	370	61
Nivolumab and refractory and relapsed Hodgkin Lymphoma	135	22

Table 2 demonstrates the results of the search strategy using MeSH terms.

**Table 2:** Database search results using MeSH terms search strategy. MeSH: medical subject headings

MeSH terms	Total article	Total articles after application of inclusion/exclusion criteria
(“Immunotherapy/therapeutic use”[Mesh]) AND “Refractory and Relapsed Hodgkin Disease/therapy”[Mesh]	78	10
(“Brentuximab Vedotin/therapeutic use”[Mesh]) AND “Refractory and Relapsed Hodgkin Disease/therapy”[Mesh]	75	27
(“Nivolumab/therapeutic use”[Mesh]) AND “Refractory and Relapsed Hodgkin Disease/therapy”[Mesh]	68	17

Data Extraction and Evaluation

Titles, abstracts, and full texts of relevant articles were collected and scrutinized for eligibility and inclusion in the discussion. The following information was extracted from the selected articles: the year of publication, the aim of the study, and findings that mainly specialize in immunotherapy and its role management of RRHL. The quality appraisal was done using the Cochrane Risk Assessment tool and PRISMA checklist for analysis included in the systematic review. After careful analysis and quality check, only moderate-to-high-quality studies were included in the final review.

Results

Search Outcome

**Figure 1:**
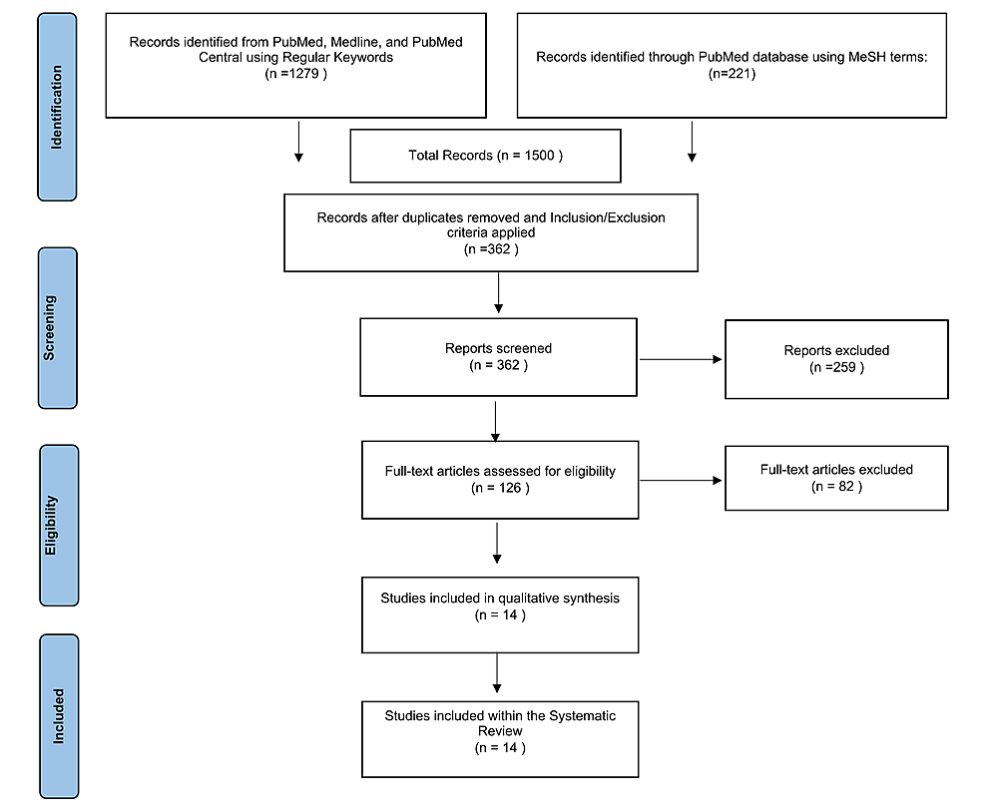
PRISMA flowchart. PRISMA: Preferred Reporting Items for Systematic Review and Meta-analysis; MeSH: medical subject headings

Using regular search keywords and MeSH terms, 1,500 articles were identified using the PubMed, PubMed Central, and Medline databases. These studies were scrutinized based on the eligibility criteria, and duplicates were removed. The remaining 362 studies were further screened manually through their titles and abstracts to determine their relevance to the focus of this study, which excluded 259 articles. Subsequently, 126 articles were assessed for eligibility. After the quality appraisal, 14 articles were included in this systematic review. Figure 1 shows the PRISMA flowchart to demonstrate the search strategy.

A total of 14 peer-reviewed studies published from 2016 to 2021 with free full texts that discussed the treatment of RRHL were included in this review. They included retrospective studies (n = 3), randomized control trials (n = 9), a case report, and a case series with moderate-to-high quality based on careful analysis and quality check. The findings of these studies are summarized in Table 3.

**Table 3:** Summary of findings. BV: brentuximab vedotin; NV: nivolumab; RRHL: refractory and relapsed Hodgkin lymphoma; ASCT: autologous stem cell transplantation

Authors, year	Study design	Purpose of study	Results
Chen et al., (2016) [13]	Phase 2 study	Assess the durability results of BV in patients with RRHL	A subset of patients who remained in complete remission for more than five years after BV monotherapy may be cured
Özbalak et al., (2019) [14]	Retrospective study	Retrospective analysis of 58 patients with RRHL treated with BV focusing on long-term remission	
Abuelgasim et al., (2019) [15]	Retrospective study	Examine the efficacy of BV combined with ifosfamide, gemcitabine, and vinorelbine (IGEV-BV) followed by ASCT in RRHL patients	IGEV-BV, as a salvage therapy, is associated with high response rates even in heavily pre-treated patients
Garcia-Sanz et al., (2021) [4]	Phase 1/2 study	Assess the efficacy and safety of BV combined with etoposide, methylprednisolone, cytosine arabinose, and cisplatin (BRESHAP) followed by ASCT in RRHL patients	BRESHAP is an effective pre-transplant induction regimen with a low toxicity profile
Iannitto et al., (2020) [16]	Retrospective study	Examine the efficacy of BV combined with bendamustine (Be-BV) in RRHL patients	Be-BV is an effective third-line treatment or pre-transplant induction regimen with a manageable toxicity profile
LaCasce et al., (2018) [17]	Phase 1/2 study	Evaluate the activity and safety of BV plus bendamustine (Be-BV) as first-line salvage regimen in RRHL	Be-BV, as the first salvage therapy in RRHL, is highly active with a manageable toxicity profile
Hwang et al., (2017) [18]	Case Report	Assess the response of low-dose NV in a patient with refractory HL	Low-dose NV induced complete remission without complications
Armand et al., (2018) [19]	Phase 2 study	Assess the safety and efficacy of NV in RRHL patients who failed ASCT	NV has long-term benefits with a favorable safety profile
Marayuma et al., (2020) [20]	Phase 2 study	Assess the safety and efficacy of NV in Japanese patients with RRHL	NV is effective and tolerable in RRHL patients. However, late-onset pulmonary toxicity may occur
Lepik et al., (2020) [21]	Phase 2 study	Evaluate the efficacy of NV-bendamustine (NB) combination in patients after failure of NV monotherapy	NB, as a salvage therapy and a pre-transplant induction regimen is highly active with an acceptable toxicity profile
Romero et al., (2020) [22]	Case series	Evaluate the efficacy of NV-bendamustine (NB) and NV- ifosfamide, carboplatin, etoposide (N-ICE) combination in three patients with RRHL	All three patients achieved a complete response with the addition of chemotherapy and consolidation with ASCT
Herrera et al., (2017) [23]	Phase 1/2 study	Evaluate the safety and efficacy of BV-NV combination as an initial salvage therapy in RRHL patients	BV-NV is a highly active and well-tolerated initial salvage therapy
Advani et al., (2021) [24]	Phase 1/2 study		
Diefenbach et al., (2020) [25]	Phase 1 study	Evaluate the safety and activity of combinations of BV with NV or ipilimumab, or both in patients with RRHL	Each treatment regimen has a complete remission rate significantly higher than expected for BV or NV-only based treatment and an acceptable safety profile. Of note, the triple therapy group has the highest toxicity

Discussion

Immunotherapy in RRHL

Salvage chemotherapy with autologous hematopoietic stem cell transplantation is recommended in patients with RRHL. However, due to the associated risks of secondary malignancy and cardiopulmonary toxicity of the procedure, several targeted treatments against CD30 and programmed death (PD) ligands are being evaluated for RRHL [9]. Normal CD30 expression is restricted to activated B and T cells. In HL, Hodgkin Reed Sternberg (HRS) cells overexpress CD30 antigen, PD ligand 1, and PD ligand 2, resulting in the suppression of T-cell proliferation and cytokine production. According to Al-Hadidi and Lee, checkpoint inhibitors (CPIs) or BV should be used until disease progression with adverse event monitoring at the goal of achieving a negative PET-CT scan [10].

BV

BV is an FDA-approved antibody-drug conjugate (ADC) with an anti-CD30 monoclonal antibody conjugated to monomethyl auristatin E, causing microtubule disruption. It has been studied in several settings to treat RRHL as a single agent in combination with chemotherapy, in newly diagnosed patients as a first-line treatment, and as maintenance therapy after ASCT for high-risk patients. The recommended starting dose is 1.8 mg/kg every three weeks. In a multinational phase 2 study in 102 post-transplant patients with RRHL, the ORR was 75%, with a CR of 34% after 16 cycles of BV monotherapy. The patients reported manageable side effects such as peripheral neuropathy and neutropenia [11]. Although BV has an acceptable safety profile, a rare and fatal adverse drug reaction (ADR) such as pulmonary toxicity may still occur. Izutsu et al. reported a post-marketing surveillance study of BV monotherapy in 284 Japanese patients (182 with HL, 101 with systemic anaplastic large-cell lymphoma). Focusing on HL, patients had a median age of 60.5 and received 5.5 treatment cycles. The overall incidence of ADR was 76.4%, and 5.5% of the HL patients had fatal pulmonary toxicity, including interstitial lung disease, respiratory failure, and acute respiratory distress syndrome. The incidence of pulmonary toxicity was elevated in patients with the prior or current pulmonary disease [12]. A summary of the key response results of BV in RRHL is demonstrated in Table 4.

**Table 4:** Key response results of BV in RRHL. ORR: objective response rate; CR: complete response; MF: median follow-up; OS: overall survival; PFS: progression-free survival; NR: not reached; BV: brentuximab vedotin; NV: nivolumab; RRHL: refractory and relapsed Hodgkin lymphoma; ASCT: autologous stem cell transplantation; IGEV-BV: BV combined with ifosfamide, gemcitabine, and vinorelbine; BEAM: carmustine, etoposide, cytarabine, and melphalan; BRESHAP: BV combined with etoposide, methylprednisolone, cytosine arabinose, and cisplatin; Be-BV: BV plus bendamustine

Authors, year, [Reference]	Study design	Eligibility criteria	Treatment and sample size	Primary endpoint	Secondary endpoint
BV as monotherapy in RRHL					
Chen et al., (2016) [13]	Phase 2 study	With RRHL after failed ASCT	Treated with 16 cycles of BV 1.8 mg/kg once every three weeks (n = 102).	ORR = 75%; CR = 34%	MF = 35.1 months; OS = 41%
Ozbalak et al., (2020), [14]	Retrospective study	RRHL patients who received four lines of prior treatment	Treated with a minimum of two cycles of BV (n = 58)	ORR = 64%; CR = 31%	MF = 20 months; OS = 26%
BV in combination with chemotherapy in RRHL					
Abuelgasim et al., (2019), [15]	Retrospective study	>14 years old; with RRHL	Median of two cycles of IGEV-BV followed by BEAM and ASCT (n = 28)	ORR = 92.5%; CR = 70%	MF = 17 months; OS = 73.5%
Garcia-Sanz et al., (2021), [4]	Phase 1/2 study	With RRHL after first-line chemotherapy; no prior history of malignant disease; life-expectancy >3 months	Median of three cycles of BRESHAP once every three weeks (n = 66)	ORR = 91%; CR = 70% (pre-transplant); CR = 82% (post-transplant)	MF = 27 months; OS = 91%
Ianitto et al., (2020), [16]	Retrospective study	>18 years old; histologically confirmed diagnosis of classic HL; with RRHL	Median of four cycles of Be-BV (n = 47)	ORR = 79%; CR = 49%	MF = 19 months; OS = 72%
LaCasce et al., (2020), [17]	Phase 1/2 study	>18 years old; with RRHL following standard chemotherapy; patients with prior exposure to BV or Be and prior salvage therapy or radiotherapy were excluded	Treated with six cycles of Be-BV followed by ASCT and 16 cycles of BV monotherapy (n = 55)	ORR = 92.5%; CR = 73.6%	MF = 44.5 months; OS = 92%

BV as monotherapy in RRHL

Chen and colleagues reported in a phase 2 study of BV monotherapy that 9% (9 of 102) of patients with RRHL following the failure of ASCT achieved remission exceeding five years. These patients did not receive additional therapy and may potentially be cured [13].

In a retrospective analysis by Ozbalak and colleagues, 58 patients with RRHL who received four lines or any prior treatment were treated with BV monotherapy. The overall response rate was 64%, with 12% achieving complete responses. The five-year PFS was 12%. At the time of analysis, five out of 12 patients had long-term remission after achieving CR with a median PFS of six years. On the other hand, three out of five patients did not receive further treatment with a PFS of six years. The study showed that some patients might be cured with BV monotherapy [14].

BV in combination with chemotherapy in RRHL

The choice of salvage regimen in RRHL as a bridge to stem cell transplant varies among centers and physician experience. A complete metabolic response (CMR) before transplant gives a good prognosis of the outcome after transplant [15]. Pre-transplant high-dose chemotherapy such as etoposide, methylprednisolone, cytosine arabinose, cisplatin (ESHAP) has an ORR of 75% and CR of 50%. A recent study that combined BV with chemotherapy reported higher responses than chemotherapy alone. A combination of ESHAP and BV as a pre-transplant regimen was evaluated to improve the CR rate before ASCT. Garcia-Sanz et al. reported an OR of 91%, with 70% achieving a complete response in 66 patients with relapsed or refractory disease treated with three cycles of BV, etoposide, solumedrol, high-dose cytosine arabinose, and cisplatin (BRESHAP) followed by ASCT and three courses of BV post-transplant. In addition to a high complete remission rate and low toxicity, the regimen has a PFS of 71% at 2.5 years [4].

Abuelgasim and colleagues reported in a retrospective study that the incorporation of BV to gemcitabine salvage regimen ifosfamide, gemcitabine, and vinorelbine (IGEV-BV) resulted in a 70% CMR rate and a 51% PMR rate. There were 28 patients with a median age of 24 years who received one to two cycles of IGEV-BV. After achieving a CMR or PMR, patients received carmustine, etoposide, cytarabine, and melphalan (BEAM), followed by ASCT. The common hematologic toxicities were neutropenia and thrombocytopenia. Eighteen patients received consolidative BV post-transplant due to disease relapse within 12 months. Post-transplant, the estimated two-year PFS was 87.1%, with an OS rate of 73.5% [15].

Bendamustine is an alkylating agent that can induce CD30 upregulation. Therefore, it can boost the sensitivity to the cytotoxic effects of BV. Iannitto and colleagues administered a median of four cycles of bendamustine in combination with BV (Be-BV) to 47 patients with previously treated RRHL. The ORR was 79%, and 49% achieved a CR. Overall, 67% of responding patients and two patients with stable disease underwent stem cell transplants and received maintenance treatment with BV alone. At a median follow-up of 19 months, the overall survival rate was 72% [16]. Of 55 patients treated with six cycles of Be-BV by LaCasce and colleagues, the ORR was 92.5%, with 73.6% achieving a CR. There were 31 patients who received BV monotherapy after Be-BV. The two-year PFS was 69.8% for post-transplant patients and 62.6% for those who did not undergo transplant after a median follow-up of 20.9 months [17]. In both studies, the Be-BV regimen was considered well tolerated with manageable toxicities but with a risk of viral reactivation. Hence, careful monitoring and antiviral prophylaxis are recommended.

NV

NV is a checkpoint inhibitor that prevents the interaction of the PD receptor and its ligands, PD-L1, and PD-L2. The FDA approved it for RRHL following auto-HSCT and BV treatment or after failing more than three lines of therapy [10]. Table 5 shows a summary of the key response results of NV in RRHL.

**Table 5:** Key response results of NV in RRHL. ORR: objective response rate; CR: complete response; MF: median follow-up; OS: overall survival; PFS: progression-free survival; NR: not reached; BV: brentuximab vedotin; NV: nivolumab; RRHL: refractory and relapsed Hodgkin lymphoma; ASCT: autologous stem cell transplantation

Authors, year, [Reference]	Study design	Eligibility criteria	Treatment and sample size	Primary endpoint	Secondary endpoint
NV as monotherapy in RRHL					
Hwang et al., (2017) [18]	Case report	A 33-year-old man underwent eight cycles of ABVD, three cycles of DHAP, ASCT with immune thrombocytopenic purpura (ITP)	The patient was given eight cycles of NV 0.55 mg/kg every two weeks	Low-dose NV-induced CR in refractory HL	
Armand et al., (2018) [19]	Phase 2 study	>18 years old; histologically confirmed diagnosis of classic HL; with RRHL after treatment failure with auto-HCT; patients with prior exposure to NV and radiotherapy within 21 days were excluded	NV with a dose of 3 mg/kg every two weeks until disease progression or unacceptable toxicity. Cohort A: BV-naïve (n = 63), B: BV received after auto-HCT (n = 80), C: BV received before and/or after auto-HCT (n = 100)	ORR = 69%; CR = 16%	MF = 18 months; OS = 92%
Marayuma et al., (2020) [20]	Phase 2 study	>20 years old; with RRHL after treatment failure with ASCT and BV	A median of 24 cycles of NV 3 mg/kg every two weeks was given until disease progression or unacceptable adverse event (n = 16)	ORR = 87.5%; CR = 35.7%	MF = 38.8 months; OS = 80.4%
NV in combination with chemotherapy in RRHL					
Lepik et al., (2020) [21]	Phase 2 study	>18 years old; histopathology confirmation of classic HL; relapsed or refractory to at least two lines of previous therapy, including treatment with NV	NV (3 mg/kg) on D1, 14 and bendamustine (90 mg/m^2^) on D1, 2 of a 18-day cycle for up to three cycles (n = 30)	ORR = 87%; CR = 57%	MF = 25 months; OS = 96.7%
Romero et al., (2021) [22]	Case series	Three patients with classic HL refractory to single-agent NV and treated with a combined approach, consisting of adding chemotherapy to nivolumab as a bridge to allo-SCT	1: A 19-year-old woman received six cycles of NV 3 mg/kg every two weeks and bendamustine 90 mg/m^2^ on days 1 and 2 of a 21-day cycle	After 27 months of allo-SCT, she is still in CR	
			2: A 35-year-old man received two cycles of NV and ifosfamide, carboplatin, etoposide (ICE) regimen	After 8 months of allo-SCT, he is still in CR	
			3: A 30-year-old man received three cycles of NV and ICE regimen	After 7 months of allo-SCT, he is still in CR	

NV as monotherapy in RRHL

Hwang and colleagues reported complete remission in a 33-year-old patient with refractory lymphoma after eight cycles of low-dose NV (0.55 mg/kg). The observation, in this case, showed that some patients require a small dose to achieve a CR with tolerable adverse effects [18].

In a multicenter phase 2 study, 243 patients with biopsy-confirmed RRHL after treatment failure with auto-HCT were divided into three cohorts: (A) patients with no prior BV treatment, (B) patients who received BV after the failure of auto-HSCT, and (C) patients who received BV before and after auto-HSCT. Patients received nivolumab 3 mg/kg every two weeks until disease progression of >10% tumor burden. Of note was the median age of 34 years; 206 (85%) of 243 patients had more than three prior lines of treatment. The CR was 16%, and the partial response was 53%, with an ORR of 69%. With a median follow-up of 18 months, the OS rate was 92%. In total, 105 patients received a median of additional eight doses of NV. The regimen was well tolerated, with few reports of fatigue (23%), diarrhea (15%), and infusion-related reactions (14%) [19].

NV can induce prolonged remission in the majority of patients. In a recent study with a long follow-up of 38.8 months, NV was administered with a median of 24 cycles in 16 patients. Pulmonary toxicities such as interstitial lung disease and pneumonitis occurred in five patients at 4.2, 17.0, 27.9, and 40.0 months. This observation necessitates long-term monitoring of patients on NV [20].

NV in combination with chemotherapy in RRHL

To increase the efficacy of immunotherapy, chemotherapy or another targeted therapy agent can be added. Anti-PD-1 therapy such as NV may restore chemosensitivity and improve anti-tumor response in patients with RRHL. Chemosensitization was demonstrated by Lepik et al. in a prospective analysis of the efficacy of nivolumab-bendamustine (NB) combination in patients after failure of NV therapy. Of 30 patients treated with the combination for relapsed or refractory disease, the ORR was 87%, with 57% achieving CR. The OS was 96.7% [21].

In a more recent study, Romero and colleagues reported three cases of heavily pretreated classic HL, refractory to NV monotherapy. All three patients achieved a CR with the addition of chemotherapy and consolidation with ASCT [22].

BV + NV in RRHL

The tumor microenvironment is an essential factor in the survival of the primary lymphoma tumor cells. HRS cells overly express CD30 and PD ligands such as PD-L1 and PD-L2 due to alteration in chromosome 9p24.1 [10]. By depleting the CD30 and PD-L-expressing HRS cells and activating the T-effector cells, therapeutic resistance can be overcome. BV and NV have an ORR of 72% and 73%, respectively, as single agents. A combination of BV and NV demonstrated an ORR of 82% and a CR of 61% in patients with RRHL. Herrera and colleagues administered four cycles of combination treatment followed by ASCT to heavily pretreated patients at a median age of 36 years. The patient tolerated the regimen and proceeded with ASCT [23].

In a more recent study, Advani and colleagues reported a phase 1/2 study in 93 patients with RRHL with a median age of 34 years and a median follow-up of 34.3 months. In total, 91 patients completed four cycles of BV and NV, and 74% proceeded to stem cell transplantation. The ORR was 85%, with a 67% complete metabolic response. The three-year PFS was 77% for all patients and 91% for those who underwent ASCT after treatment. The three-year OS rate was 93%. These data prove that a combination of BV and NV can be used as a non-chemotherapeutic approach as a bridge to transplant [24].

Diefenbach and colleagues conducted a phase 1 study on the efficacy of the combination of BV with ipilimumab (ipilimumab group), NV (nivolumab group), and both NV and ipilimumab (triple therapy group) on 61 patients with RRHL aged 18 years or older who had relapsed after at least a single line of therapy. All cycles were 21 days apart with a median of seven cycles and a maximum duration of one to two years. The ORR was 76% in the ipilimumab group, 89% in the NV group, and 82% in the triple therapy group, with a CR of 57%, 61%, and 73%, respectively. The median PFS was not reached except in the ipilimumab group (1.2 years) [25]. Currently, the tolerability and activity of the NV group and the triple therapy group are being compared in a phase 2 trial. With a long-term follow-up of more than two years, patients had sustained remission in the absence of hematopoietic cell transplantation. The phase 3 study will evaluate the effective regimen if it can become a second-line therapy as a bridge to transplantation or forego hematopoietic cell transplantation. A summary of the key response results of BV + NV in RRHL is demonstrated in Table 6.

**Table 6:** Key response results of BV + NV in RRHL. ORR: objective response rate; CR: complete response; MF: median follow-up; OS: overall survival; PFS: progression-free survival; NR: not reached; BV: brentuximab vedotin; NV: nivolumab; RRHL: refractory and relapsed Hodgkin lymphoma

Authors, year, [Reference]	Study design	Eligibility criteria	Treatment and sample size	Primary endpoint	Secondary endpoint
BV + NV in RRHL					
Herrera et al., (2017) [23]	Phase 1/2 study	>18 years old; With RRHL following standard chemotherapy; patients with prior immune-oncology therapy and prior salvage therapy or radiotherapy were excluded	Median of four cycles of BV-NV (n = 62)	ORR = 82%; CR = 61%	MF = 7.8 months; six-month PFS = 89%
Advani et al., (2021) [24]	Phase 1/2 study	>18 years old; with RRHL following standard chemotherapy; patients with prior exposure to BV or Be and prior salvage therapy or radiotherapy were excluded	Median of four cycles of BV-NV (n = 91)	ORR = 85%; CR = 67%	MF = 34.3 months; OS = 93%
Diefenbach et al., (2020) [25]	Phase 1 study	>18 years old; with RRHL following standard chemotherapy	BV + ipilimumab (n = 23)	ORR = 76%; CR = 57%	MF = 2.6 years; PFS = 1.2 years
			BV + nivolumab (n = 19)	ORR = 89%; CR = 61%	MF = 2.4 years; PFS = NR; OS = NR
			BV + ipilimumab + nivolumab (n = 22)	ORR = 82%; CR = 73%	MF = 1.7 years; PFS = NR; OS = NR

Limitations

Studies included in this review were limited to a specified time frame in the last five years. It is important to note that the results of the studies conducted before 2016 may also be valuable in the management of RRHL. To assess the durability of response, an extended follow-up is required.

## Conclusions

Therapeutic resistance can be overcome in RRHL by depleting the CD30 and PD ligand expressed by Hodgkin Reed Sternberg cells using immunotherapy. The utilization of BV and NV as a single agent or in combination with chemotherapy as salvage therapy for RRHL produced a high ORR and CR rate. The ORR and survival rates of BV + NV combination therapy were higher than those observed with BV or NV as single agents in RRHL. Consequently, the combination of BV + NV can be an alternative salvage regimen to chemotherapy as a bridge to stem cell transplant. Furthermore, the adverse effects are tolerable with a good safety profile.

A majority of the patients were able to undergo ASCT, and patients who proceeded to ASCT had a more favorable PFS and OS. The review confirms the durable response and good safety profile as a salvage regimen with sustained benefits in young and older patients, especially with comorbidities.
